# Retraction: Clinical Significance, Cellular Function and Potential Molecular Pathways of CCT7 in Endometrial Cancer

**DOI:** 10.3389/fonc.2020.641611

**Published:** 2021-01-14

**Authors:** Frontiers Editorial Office

The journal retracts the 28^th^ August 2020 article cited above.

Following publication, concerns were raised regarding the integrity of Figures 1 and 3. An investigation was conducted in accordance with Frontiers guidelines and the authors failed to provide a satisfactory explanation.

This retraction was approved by the Chief Editors of Frontiers in Oncology and the Editor-in-Chief of Frontiers. The authors agree to this retraction.

